# Development of a Digital Photographic Food Atlas as a Portion Size Estimation Aid in Japan

**DOI:** 10.3390/nu14112218

**Published:** 2022-05-26

**Authors:** Nana Shinozaki, Kentaro Murakami, Keiko Asakura, Shizuko Masayasu, Satoshi Sasaki

**Affiliations:** 1 Department of Social and Preventive Epidemiology, School of Public Health, The University of Tokyo, 7-3-1 Hongo, Bunkyo-ku, Tokyo 113-0033, Japan; nana-s@m.u-tokyo.ac.jp (N.S.); stssasak@m.u-tokyo.ac.jp (S.S.); 2 Department of Environmental and Occupational Health, School of Medicine, Toho University, 5-21-16 Omori-Nishi, Ota-ku, Tokyo 143-8540, Japan; keiko.asakura@med.toho-u.ac.jp; 3 Ikurien-Naka, 3799-6 Sugaya, Ibaraki 311-0105, Japan; sizuko-masa@themis.ocn.ne.jp

**Keywords:** food atlas, photograph, digital image, dietary record, portion size, Japan, dietary assessment

## Abstract

This study aimed to develop a digital photographic food atlas as a portion size estimation aid. Commonly consumed foods were identified from the 5512-day weighed dietary records of 644 Japanese adults. Portion sizes were determined based on the market research and distribution of food consumption in the dietary records. Each food item was classified into one of two photo types: a series of photographs showing gradually increasing portion sizes or guide photographs representing a range of portion sizes and food varieties in one photograph. Photographs of the food were taken at an angle of 42°, along with appropriate reference objects such as chopsticks. In total, 209 food and dish items were included in the food atlas. Series of photographs were taken for 105 items that are not usually served in predetermined amounts (e.g., rice and pasta), whereas guide photographs were taken for 104 items usually served in predetermined amounts (e.g., bananas and cookies). Moreover, photographs were taken for 12 kinds of household measurement items, such as cups and glasses. The food atlas could be a valuable tool for estimating the portion size in dietary surveys. Evaluating the validity of this food atlas for portion size estimation is warranted.

## 1. Introduction

Portion size estimation is an essential component of dietary assessment [1]. Misestimation of portion size may increase estimation errors of food and nutrient intake, potentially causing a misinterpretation of the association between diet and health outcomes [1]. Although weighing foods is considered a gold standard to assess food amounts, it is time-consuming and requires high motivation from participants [1,2]. This hinders the use of weighing methods in large-scale or long-term dietary surveys [1,2,3].

Therefore, various portion size estimation aids have been developed, including household utensils, food models, and food photographs [4]. Food photographs are widely used in dietary surveys because they are less burdensome for respondents. Moreover, they are easy to use for interviewees, portable and inexpensive, and cover a broad range of foods [4,5,6,7]. During data collection, respondents can report the amounts of foods they consumed by selecting a single picture or indicating a fraction, multiple, or percentage of the amount shown in a photograph [8]. A previous review suggested that food atlases (a photo album of various food portion sizes) are more accurate than a limited number of photographs in other formats [4].

Food atlases must be developed on a country-by-country basis, based on food availability and dietary intake data [3,6]. Presently, food atlases or comprehensive food photograph databases have been developed in many countries in Europe [3,6,8,9,10,11,12,13,14,15,16,17], North and South America [5,18,19,20,21,22], the Middle East [23,24], Africa [25,26,27], and Asia [28,29,30,31,32,33]. However, in Japan, there is no comprehensive food atlas that has been developed using a data-driven approach. For future dietary surveys, it is essential to develop a food atlas that encompasses a wide range of foods and dishes consumed in Japan based on dietary intake data. Therefore, this study aimed to develop a digital photographic food atlas to help estimate portion size in dietary surveys in Japan.

## 2. Materials and Methods

### 2.1. Data Source

The development of the food atlas was based on weighed dietary data derived from two surveys. Since both the surveys have been described previously [34,35], only a brief explanation has been provided here. The first survey was conducted between 2002 and 2003 in four prefectures in Japan (2003 survey), and the second one was conducted in 2013 in 23 prefectures (2013 survey). The 2003 survey participants included healthy females (*n* = 126) aged 30–69 years and their cohabitating spouses (*n* = 126), while the 2013 survey included healthy females (*n* = 196) and males (*n* = 196) aged 20–69 years. In total, 644 participants provided their dietary data. A weighed dietary record (DR) was collected over 16 days (three weekdays and one weekend day in each of the four seasons) in the 2003 survey and for 4 days (three working days and one non-working day during the winter season) in the 2013 survey. Local registered dietitians used written and verbal instructions to explain to the participants how to complete the DR using recording sheets and a digital scale (KD-173 (2003 survey) and KD-812WH (2013 survey), Tanita, Tokyo, Japan). Recording sheets were checked by the registered dietitians at the local center and then again at the study center. The registered dietitians coded each food in the “food item” column of the recording sheet at the study center, following uniform procedures using food codes from the Standard Tables of Food Composition in Japan [36,37]. Estimated intakes of energy and nutrients from each food item were computed based on the intake of food items and their nutrient contents [38]. Moreover, each dish in the “dish item” column in the DR was coded using the dish codes of a recently developed dish composition database in Japan [39,40]. In the 2003 survey, 10 participants provided less than 16 days of the DR: 4 days (*n* = 2) and 8 days (*n* = 8). Consequently, the dataset comprised 5512-day dietary data (3944 days in the 2003 survey and 1568 days in the 2013 survey), including 1591 food codes and 371 dish codes.

### 2.2. Selection of Foods and Dishes

**Figure 1:**
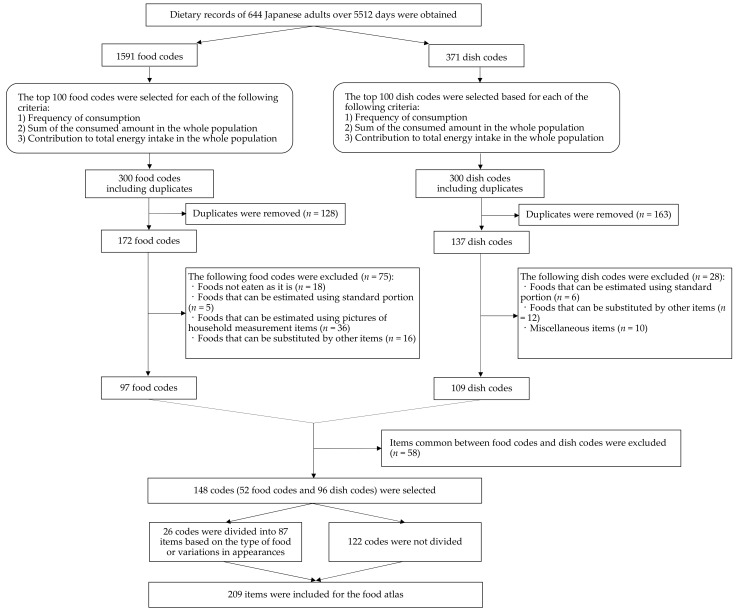
Selection flow of the foods and dishes for the food atlas.

We systematically selected commonly consumed foods and dishes for the food atlas (Figure 1). First, the frequency of consumption, sum of the consumed amount, and energy contribution in the entire population were calculated for each food code and dish code [10,41]. Following this, the top 100 food codes and top 100 dish codes were identified for each criterion, resulting in 300 food codes and 300 dish codes chosen, including duplicates. After eliminating the duplications from the union of the top 100 items for each criterion, 172 food codes and 137 dish codes remained. Next, we excluded the following items that did not require a photograph for portion size estimation:

1. Foods not eaten as they are (e.g., flour, starch);
2. Items that can be estimated using standard portions without food photographs (e.g., boiled eggs, candy);
3. Items that can be estimated by pictures of household measurement items (e.g., soy sauce can be estimated by tablespoons);
4. Items that can be substituted by other items consumed more commonly (e.g., the food code 17042 “mayonnaise, whole egg type” can be substituted by the food code 17043 “mayonnaise, egg yolk type”) [39];
5. Miscellaneous dish codes (e.g., dish code 11522 “beverages [details unknown]”) [39].

After excluding 75 food codes and 28 dish codes, 97 food codes and 109 dish codes were retained. We excluded 58 items common between the food codes and dish codes (e.g., food code 16011 “fermented alcoholic beverage, wine, red” was excluded to avoid duplication with dish code 11517 “wine”). Finally, 148 codes (52 food codes and 96 dish codes) were selected for inclusion in the food atlas.

After the list of food and dish codes was defined, we selected a specific food and dish item to purchase for each code based on the DR. For dish codes consisting of similar types of multiple dishes, we selected the dish that appeared most frequently in the DR (e.g., for dish code 10105 “curry and rice/hash and rice,” we selected curry and rice). If there were several distinctive items in a single code, we divided the code into subdivisions for clarity of classification. For instance, dish code 10308 “yakisoba” (stir-fried noodles with vegetables and meat) was divided into “packaged ready-to-eat yakisoba” and “yakisoba on a plate.” Similarly, if a food item had a large variation in appearance, food packaging, and serving method, the food code was divided into certain subdivisions. In this way, 26 codes were divided into 87 items (i.e., 2–11 subdivisions for each code) based on the type of food or variations in appearances. The remaining 122 codes were not divided. Consequently, 209 food and dish items (including 87 items that are variations of 26 codes and 122 items without variations) were included in the food atlas. Based on the DR, we determined how to cut and prepare each food item to represent the state in which it is usually eaten. Food and dishes were purchased from popular restaurants, local and online supermarkets, and convenience stores.

### 2.3. Determination of the Type of Photographs

**Figure 2:**
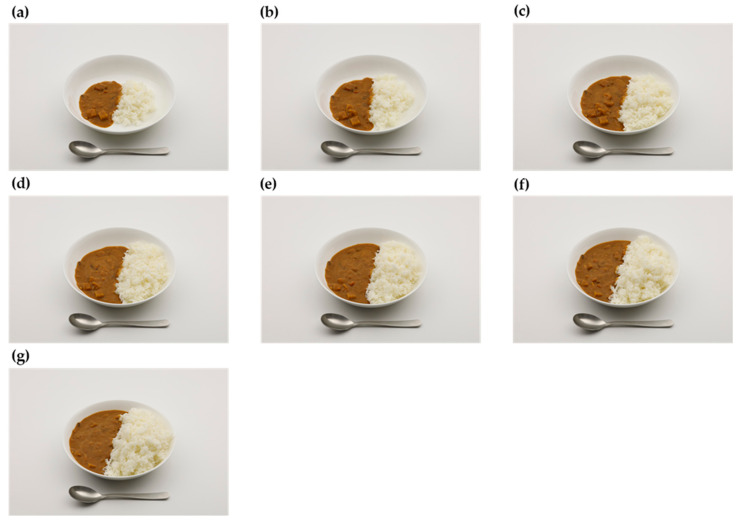
Example of a series of photographs for curry and rice. The portion size gradually increases from photo (**a**–**g**).

**Figure 3:**
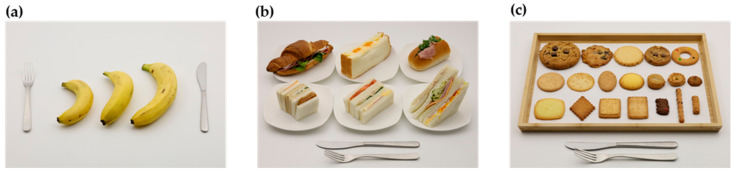
Examples of guide photographs. (**a**) Bananas, (**b**) sandwiches, and (**c**) cookies.

Based on previous studies [10,24,29,41], we decided to take photographs of the foods and dishes either as a series of photographs (Figure 2) or guide photographs (Figure 3). A series of photographs depicts gradually increasing portion sizes for foods and dishes that are usually not served in predetermined amounts (e.g., white rice, curry and rice, pasta, and vegetable salads). A guide photograph represents a range of the common portion sizes in one photograph for foods and dishes that are usually served in predetermined amounts (e.g., bananas, cookies, doughnuts, and milk in cartons).

We classified each food and dish item into either photograph type. If a food item could be represented in both types of photographs, then the photograph type was selected according to the variability of sizes in the market, portion size distribution in the DR, and authors’ knowledge.

**Figure 4:**
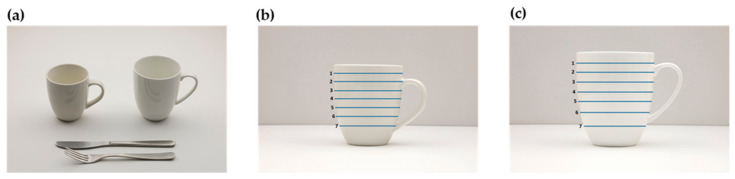
Examples of photographs of household measurement items. (**a**) An image to select the size of corn-shaped mugs and (**b**,**c**) images to select portion size of the left and right mugs, respectively, from photo (**a**).

In addition to the series of photographs and guide photographs, we took photographs of household measurement items as options to estimate the portion size of beverages and seasonings (Figure 4). These household measurement items included various cups, glasses, meal spoons, and measuring spoons.

### 2.4. Determination of Portion Sizes

We determined the portion size of each food and dish according to the type of photograph. For guide photographs, we purchased various products from popular local and online supermarkets, where we selected items that represent a variety of portion sizes and the appearance of each food and dish item in a single photo.

The number of portion sizes in a series of photographs was set as seven [10,41]. The first (smallest) and seventh (largest) portion sizes were the 5th and 95th percentiles of the weight consumed in the DR, respectively [8,10,42]. The second to sixth portion sizes were determined based on equal increments on a log scale from the 5th to the 95th percentile of the weight of food consumed in one eating session, allowing the portions to increase proportionately [10,24]. For dishes consisting of food ingredients with different physical characteristics, such as curry and rice, the portion of each part was determined according to the commercial products or common recipes. For instance, for curry and rice, the portions of white rice and curry sauce were determined separately based on the average ratio of white rice to curry sauce calculated from two beef curry dishes purchased from a popular restaurant chain (CURRY HOUSE CoCo Ichibanya). Foods usually eaten after heating (raw vegetables and mushrooms) were microwaved as appropriate to obtain the yield factor (i.e., the rate of weight change before and after cooking). Each portion size was then calculated as the weight of the raw ingredient for each portion multiplied by the yield factor.

For photographs of cups and glasses, the number of portion sizes was set as five or seven based on the size of the cups or glasses. The maximum portion size was defined as the maximum volume (1 cm from the edge) of each cup or glass. Subsequently, the maximum volume divided by the number of portion sizes (five or seven) was used as the increase in each portion from the first to sixth portion sizes.

### 2.5. Preparation of Tableware

Tableware was selected from popular tableware stores in Japan (Nitori Co. Ltd., Ikea Japan K.K., Ryohin Keikaku Co. Ltd., Tokyo, Japan). To make it easy to see the color and shape of foods, we chose plain white plates, except for a wooden bowl or tray for foods that were white in color, such as grated radish. Furthermore, because rice, soup, and beverages are frequently consumed using a wide variety of tableware, we selected rice bowls, soup bowls, and cups and glasses of assorted sizes and shapes. This was done so that people could estimate the portions using tableware that was similar to what they usually used.

### 2.6. Weighing of Foods

Each portion of food was weighed using a calibrated cooking scale, KW-320 (Tanita, Tokyo, Japan), measuring up to 300 g in increments of 0.1 g, 300–1500 g in increments of 0.5 g, and 1500–3000 g in increments of 1 g. In the series of photographs, each portion was adjusted to equal each portion calculated from the DR as closely as possible. The weights of the beverages were calculated by multiplying the liquid volume (mL) by the specific gravity (g/mL) of each drink item in the Standard Tables of Food Composition in Japan [38]. The weight of each portion was recorded and entered into a database.

### 2.7. Photography

To ensure the quality of food photography, the authors attended three lecture sessions by a professional food photographer. The authors (N.S. and K.M.) captured all photographs using a 26.2-megapixel camera, EOS-RP (Canon Inc., Tokyo, Japan).

We mainly used a 26-cm dinner plate, 15-cm and 11-cm side plates, 21-cm and 18-cm bowls, and a 40 × 30-cm wooden tray, as appropriate. Photos of foods and dishes were taken at an angle of 42°, which is considered an average viewing angle from a sitting position at a table and the best balance between depth and height [11,14,43]. The photographs to select rice and soup bowls and cups and glasses were taken at an angle of 30°. This angle was determined in consultation with the professional food photographer to make it easier to see the differences in tableware size. Photographs of cups and glasses for assessing the liquid volume of beverages were taken horizontally, with scale lines representing each portion added using Microsoft PowerPoint for Microsoft 365 (Microsoft, Redmond, WA, USA), as shown in Figure 4b,c. One or two standard reference objects, such as a 23-cm chopstick, 21-cm knife, 19-cm fork, 19-cm spoon, or 14-cm dessert spoon, which were considered most appropriate for each food and dish, were included in the pictures to help recognize the quantity of foods and dishes and the size of the plate [24,42]. To achieve consistent exposure and white balance, we used a shutter speed of 1/50; aperture, F10; ISO, 1600; and a grey card (GIN-ICHI Co., Tokyo, Japan). The camera was connected to a laptop computer, and the consistency in the pictures was reviewed using the software program EOS Utility, version 3.13.30 for Windows (Canon Inc., Tokyo, Japan). Lighting was provided using two softboxes (58 watts). In addition, a soft diffuser disc panel was used to diffuse the light softly. Each photograph of the food and dish item was assigned a picture ID corresponding to its original food and dish code.

## 3. Results

Of 209 food and dish items included in the food atlas, 105 were photographed as a series of photographs and 104 as guide photographs.

### 3.1. Series of Photographs

Figure 2 shows an example of a series of photographs of curry and rice, and Table 1 shows a list of the items in the series of photographs. Most items had seven portions, whereas white rice and miso soups had up to three to six portions for small- and medium-sized bowls, because there was no capacity to serve large portions.

### 3.2. Guide Photographs

Examples of the guide photographs are shown in Figure 3, and the list of foods is presented in Table 2. The number of portions selected in a single photograph varied according to the food and dish items, ranging from 2 to 19 (mean: 5.3). We decided to use the same photographs of paper cartons, plastic bottles, or cup bottles for different beverages (e.g., soymilk, milk, and vegetable juice), because these containers are common for various kinds of beverages. Protein and energy bars were photographed due to their increasing popularity, although they did not appear frequently in the DR.

### 3.3. Household Measurement Photographs

A summary of the household measurement items photographed is presented in Table 3. In total, 12 kinds of household measurement items were included in the food atlas. The number of portions was five or seven according to the size of the cups or glasses. As shown in Figure 4, scale lines indicating each portion were added to each cup and glass. However, the portion size of tokkuri (Japanese sake bottle) and choko (small cup for Japanese sake) was set as only the maximum volume, because it was difficult to measure different volumes of liquid in opaque containers. Moreover, they are usually served in 1-cup units. Similarly, the portion size of only the maximum volume is available for meal spoons and measuring spoons.

## 4. Discussion

To our knowledge, this is the first comprehensive food atlas designed based on Japanese adult dietary intake data. Although a food atlas was recently published and used in a 24-h dietary recall method in Japan, no information was provided on the data source, development process, or the types and number of photographs [44]. In contrast, our food atlas was developed using multiple day weighed DRs, and includes a detailed description of its development process and structure. Our food atlas includes 209 commonly consumed food and dish items. Of these, 105 and 104 are presented as a series of photographs, and as guide photographs, respectively. Moreover, our food atlas comprises photographs of 12 kinds of household measurement items, including cups and glasses. This food atlas could be a valuable tool to help estimate portion sizes in dietary surveys in Japan.

It is important to use population-based weighed dietary data to determine the type of foods and ranges of portion sizes to be included in a food atlas [42]. Most previous studies have used population-based dietary data to develop food photographs [3,5,8,10,12,16,17,20,23,24,27,29,32,45,46]. However, some studies have relied on other sources, such as interviews and focus group discussions [25,26], researchers’ observations [47], and experiences of investigators or dietitians [13,14]. This study used the data from the weighed DRs collected over four seasons among Japanese adults. This enabled us to include items representing Japanese dietary habits with day-to-day and seasonal variations in the food atlas.

There is no gold standard for which foods and how many foods should be included in a food atlas. Food atlases should comprise foods with various portion sizes and irregular shapes and sizes [42]. Meanwhile, it is unnecessary to include foods that are readily identifiable from the description (e.g., packaged foods). Including such foods makes the food atlas more expensive and portion size estimation becomes more time-consuming [42]. Therefore, we set inclusion and exclusion criteria for foods and dishes and systematically selected popular and essential items. The number of foods and dishes comprised in this atlas (*n* = 209) is comparable to that of previously validated food atlases [8,23]. Moreover, if food types and physical attributes are similar, then some photographs may be available to estimate the portion size of other food items not listed in the atlas. In such cases, an evaluation of food appearance and weight adjustments based on food density would be necessary [8,42].

Food atlases should be designed to minimize error in estimating food portion sizes, which is affected by the format of the photographs, such as the number and range of portion sizes depicted [42]. The number of potion sizes per food item varied among previous studies, ranging from one [43,48,49] to eight [18,24,29,43,45]. There is no standard for how many photographs should be shown for a food item [25,43]. However, it has been reported that portion size estimation using one portion photograph was less accurate than estimation using eight photographs [43]. Another study indicated that four images provided less accurate estimates than eight images, albeit not to a statistically significant extent [19]. This may be because respondents may have difficulty in estimating fractions or multiples of portion size, and fewer photographs lead to a loss of precision [42]. In this study, we chose seven portion sizes for a series of photographs of most items based on previous studies [10,41]. Moreover, as previously recommended [42], we determined the range of the food portions systematically by selecting the 5th to 95th percentiles of the amounts of foods consumed. This helps depict very small and very large portion sizes, making the food atlas suitable for use in populations with variations in the amount of food eaten [42]. The interval of portion sizes depicted between images is also important [42]. We decided to determine portion sizes in a series of photographs based on a log scale so that each portion increased proportionately [19]. For foods depicted as guide photographs, we selected various items based on observations of products at popular local and online supermarkets. This would be helpful to represent in the photograph a range of portion sizes that people typically consume.

The size of photographs also influences estimation error [42]. Although no consistent associations have been observed between the size of photographs and estimation accuracy [19,28,43,50], participants tend to prefer larger photographs to smaller ones [19]. As our food atlas is digital, the photographs can be shown or printed in any size. Previous studies have shown that printed and digital photographs provided similar estimates [5,51]. It is also unclear whether photographs should be labeled with portion size information, such as grams or cups. No existing studies have assessed if labelling will improve accuracy or if it causes bias leading to misreporting [19,52]. The photographs in our food atlas were not labeled to ensure their flexible use. It would be helpful to evaluate the effects of food weight labeling on photographs in future studies. 

In this food atlas, each photograph has information regarding weights; therefore, it can help to estimate the amount of consumed food by selecting a specific photograph or reporting the relative portion size to that depicted in the photograph. Given that the food atlas contains photographs of various foods and dishes commonly consumed in Japan, it is potentially helpful in other countries to estimate Japanese food intake, although portion size distribution should be carefully considered. We can also calculate energy and nutrient intake from the food photographs, because each food and dish item can be connected to the Standard Tables of Food Composition in Japan [38] and the dish composition database [39,40]. In addition, because food photographs are digital data, they can be displayed on various devices (e.g., tablets or laptops) or printed. Thus, the food atlas can be used in combination with various dietary assessment methods, such as both paper-based and web-based FFQs, 24-h recalls, and DRs. However, as a prerequisite for using the food atlas in dietary surveys, the accuracy of dietary intake estimated using the food atlas should be evaluated first.

The strength of this study is the use of DR data obtained over multiple days during four seasons to develop the food atlas. This enabled a data-driven approach to select food and dish items and determine portion sizes, resulting in a comprehensive database of food photographs in Japan. Moreover, each development process was based on scientific principles and practical experience. Nevertheless, this study had several limitations. First, since the dietary data used in this study were obtained from healthy volunteers who were not randomly selected, it may not represent the types and quantities of foods typically consumed in Japan. Food atlases would be more representative if developed based on dietary data from national food consumption surveys [5,50]. However, we did not use the Japanese national dietary survey data because they employ a single-day, household-level DR, and cover only 1 month of the year [53]. It thus seemed inadequate for investigating the portion sizes of various foods. Nevertheless, the weight and height of the participants in this study were similar to those of the general Japanese population [54]. Second, the 2013 survey only included the dietary data obtained in the winter season. This may hinder capturing food eaten in other seasons due to a seasonal variation in dietary intake among Japanese adults [55,56,57]. Moreover, since the two dietary data used were relatively old, the types and amounts of foods included in the food atlas may not reflect the current diet of the Japanese population. Given that the dietary habits of Japanese people have been gradually changing [58], the foods depicted in the atlas should be updated according to people’s dynamic eating habits and market composition. Third, because the food atlas was developed based on dietary data obtained only from adults, it may not be used to estimate dietary intake in children. A previous study emphasized the need for age-appropriate food photographs to improve the quality of dietary intake data collected from children [59]. Fourth, self-reported dietary data are subject to misreporting [60], leading to errors in portion sizes calculated from the DR. Therefore, further assessment is needed to confirm whether the food atlas encompasses the relevant portion sizes among Japanese people. Fifth, the selection of types and portion sizes of foods and dish items, and the determination of the photographic presentation of each item, involved some subjective judgments. The authors carefully assessed dietary data, conducted market research, and scrutinized recipe books to make the selection and appearance of food and dish items as general as possible. Finally, the food atlas has not yet been evaluated for its validity in estimating portion size. Portion size estimation aids should be validated in the target population [6]. We have completed the validation study in a Japanese population to assess the accuracy of portion size estimates using the food atlas. The differences between the amount of food estimated from the food atlas and the actual food weight are currently under evaluation.

## 5. Conclusions

We developed a digital photographic food atlas for Japanese adults based on DR data and provided a detailed explanation of each procedure. The food atlas contained 209 commonly consumed food and dish items among Japanese adults and 12 kinds of household measurement items, such as cups and glasses. The food atlas would be a practical and easy-to-use instrument to help estimate portion size both for researchers and respondents. Further research is needed to evaluate how accurately respondents can estimate food portion sizes using the food atlas.

## Figures and Tables

**Table 1:** List of items in the series of photographs (*n* = 105).

Category	Picture ID ^1^	Food/Dish Name	Number of Portions
Food item	F1039	Soba (buckwheat noodles)	7
	F1044	Somen (thin wheat noodles)	7
	F1088s	White rice in a small rice bowl	6 ^2^
	F1088m	White rice in a medium rice bowl	6 ^2^
	F1088l	White rice in a large rice bowl	7
	F1128	Soba (buckwheat noodles)	7
	F2003	Simmered konjac balls	7
	F2017	French fries	7
	F2023	Grated yam	7
	F4039	Deep-fried bean curd, chopped	7
	F4040	Fried tofu, shredded	7
	F6061	Cabbage, shredded	7
	F6065	Cucumber, sliced	7
	F6084	Burdock, sliced, microwaved	7
	F6134	Radish, shredded	7
	F6153	Onion, thinly sliced	7
	F6191	Eggplant, chopped, microwaved	7
	F6207	Chinese chive, microwaved	7
	F6214	Shredded carrots	7
	F6226	Spring onion, chopped	7
	F6233	Chinese cabbage, chopped, microwaved	7
	F6245	Green bell pepper, shredded, microwaved	7
	F6264	Broccoli, microwaved	7
	F6291	Bean sprouts, microwaved	7
	F6312	Lettuce, cut into bite-sized pieces	7
	F7012	Strawberry	7
	F7049	Persimmon, sliced	7
	F7077	Watermelon, sliced	7
	F8001	Enoki mushroom, microwaved	7
	F8016	Brown beech mushroom, microwaved	7
	F8039	Shiitake mushroom, sliced, microwaved	7
	F9045	Wakame seaweed	7
	F10070	Japanese eel	7
	F10263	Canned tuna, served in a dish	7
	F11183	Bacon, shredded	7
	F13025b	Yoghurt, served in a bowl	7
	F14020a	Margarine, served with a butter knife	7
	F14020b	Margarine, served on a slice of bread	7
	F17036	Ketchup	7
	F17039	Japanese-style dressing	7
	F17043	Mayonnaise	7
Dish item	D10104	Zousui (porridge of rice and vegetables) soup	7
	D10105a	Japanese curry and white rice	7
	D10105b	Thai green curry and jasmine rice	7
	D10109	Fried rice	7
	D10112	Beef rice bowl	7
	D10113	Pork cutlet rice bowl	7
	D10114	Oyako-don (chicken and egg rice bowl)	7
	D10116	Grilled eel and rice	7
	D10119	Chirashizushi (sushi rice topped with various raw fish pieces)	7
	D10301	Udon (thick wheat noodles) with soup	7
	D10302	Soba (buckwheat noodles) with soup	7
	D10305	Ramen	7
	D10307a	Napolitan (ketchup-based spagetti)	7
	D10307b	Penne with cheese sauce	7
	D10308a	Yakisoba (fried noodles) on a plate	7
	D10312	Soki soba (traditional noodle in Okinawa)	7
	D10401	Okonomiyaki (savoury pancake with various ingredients)	7
	D10501	Nikujaga (meat and potato stew)	7
	D10502	Simmered squid and taro	7
	D10503	Baked sweet potato	7
	D10506	Potato salad	7
	D10801	Spinach seasoned with sesame sauce	7
	D10802	Ohitashi (boiled spinach)	7
	D10803	Vinegared cucumber and wakame seaweed	7
	D10805	Grated daikon radish	7
	D10810a	Vegetable mix salad	7
	D10810b	Shrimp and broccoli salad	7
	D10810c	Bean salad	7
	D10814	Macaroni salad	7
	D10815	Vinegared mozuku seaweed	7
	D10819	Kinpira (chopped burdock root and carrot cooked in sugar and soy sauce)	7
	D10822	Simmered squash	7
	D10823	Simmered Japanese butterbur and deep-fried tofu	7
	D10826	Simmered hijiki seaweed	7
	D10835	Vegetable stir-fry	7
	D10904	Cut apples	7
	D11001a	Tuna sashimi	7
	D11001b	Tuna strips	7
	D11012	Simmered flatfish	7
	D11013	Simmered mackerel in miso	7
	D11022	Grilled salmon	7
	D11024	Grilled mackerel	7
	D11117	Chikuzenni (simmered root vegetables with chicken)	7
	D11123	Sliced beef steak	7
	D11125	Ginger fried pork	7
	D11134	Chinese stir-fry containing green peppers and meat	7
	D11141	Pork cutlet	7
	D11204	Tamago-yaki (Japanese rolled omelette)	7
	D11701s	Miso soup (with tofu and green onion) in a small soup bowl	3 ^2^
	D11701m	Miso soup (with tofu and green onion) in a medium soup bowl	6 ^2^
	D11701l	Miso soup (with tofu and green onion) in a large soup bowl	7
	D11703s	Tonjiru (miso soup with pork and vegetables) in a small soup bowl	3 ^2^
	D11703m	Tonjiru (miso soup with pork and vegetables) in a medium soup bowl	5 ^2^
	D11703l	Tonjiru (miso soup with pork and vegetables) in a large soup bowl	7
	D11711a	Creamy corn soup	7
	D11711b	Clam chowder	7
	D11714	White stew	7
	D11718	Yosenabe (seafood and vegetable hot pot)	7
	D11802a	Tsukudani of konbu seaweed (simmerd konbu)	7
	D11802b	Laver boiled in soy sauce	7
	D11805	Cubed daikon kimchi	7
	D11806	Pickled scallions	7
	D11807a	Pickled cucumber	7
	D11807b	Pickled Chinese cabbage	7

^1^ For food items, the four- or five-digit number corresponds to the food code in the Standard Tables of Food Composition in Japan [38]. For dish items, the five-digit number corresponds to minor codes in the dish composition database [39]. The alphabet at the end of each code indicates subdivisions. ^2^ Large portion sizes cannot be served in small and medium bowls.

**Table 2:** List of items in the guide photographs (*n* = 104).

Category	Picture ID ^1^	Food/Dish Name	Number of Portions
Food item	F1034	Bread roll	6
	F1088b	Pre-cooked rice	7
	F1117	Sticky rice cake	6
	F4032	Tofu	7
	F4046	Natto (fermented soybeans)	4
	F6182	Tomato	6
	F7107	Banana	3
	F10381	Chikuwa (Japanese fishcake)	3
	F11186	Sausage	6
	F13025a	Packed yoghurt	10
	F13040	Cheese	10
	F15009	Casutera sponge cake	4
	F15069	Anpan (sweet bean-jam bun)	4
	F15070	Cream bun	3
	F15076a	Croissant	4
	F15076b	Danish bread	6
	F15082a	Madeleine	3
	F15082b	Pound cake	2
Dish item	D10118	Rolled sushi	4
	D10120	Rice ball	13
	D10201	Toast	6
	D10208	Melon bread (half-melon shaped buns)	3
	D10208b	Stick bread	3
	D10209	Sausage roll	3
	D10211	Sandwich	6
	D10213	Hamburger	5
	D10304a	Instant noodle (packet)	3
	D10304b	Instant noodle (Cup Noodle-shaped container)	3
	D10304c	Instant noodle (Donbei-shaped container)	3
	D10304d	Instant noodle (other shaped container)	2
	D10308b	Yakisoba (UFO)	2
	D10308c	Yakisoba (Peyoung)	2
	D10308d	Yakisoba (other)	2
	D10510	Potato croquette	5
	D10622	Soymilk ^2^	5
	D10908	Mandarin orange	3
	D10909	Various kinds of citrus	8
	D11021	Dried fish	5
	D11046	Fish jelly products	8
	D11102	Fried gyoza dumplings	4
	D11108	Hamburg steak	5
	D11143	Deep-fried chicken	5
	D11301	Milk ^2^	5
	D11304	Coffee-flavoured or fruit-flavoured milk ^2^	5
	D11307	Lactic acid bacteria beverage	5
	D11401	Kusamochi (rice-flour dumplings mixed with mugwort, stuffed with red bean paste)	3
	D11402	Manjuu (sponge cake stuffed with red bean paste)	8
	D11412	Rice cracker	9
	D11419a	Cookie (except sandwich cookie)	19
	D11419b	Sandwich cookie	9
	D11419c	Cracker	6
	D11419d	Energy bar	6
	D11419e	Protein bar	8
	D11420a	Chocolate with nuts	6
	D11420b	Chocolate candy bar	5
	D11420c	Chocolate snack cake	4
	D11420d	Chocolate-coated biscuit stick (e.g., Pocky)	4
	D11420e	Chocolate-coated biscuit stick (e.g., Lumonde)	6
	D11420f	Chocolate-coated biscuit	12
	D11420g	Other chocolate snacks (e.g., chocolate puffs)	8
	D11420h	Chocolate bar without nuts or cookies (e.g., Ghana)	7
	D11420i	Chocolate balls without nuts or cookies (e.g., Meltykiss)	8
	D11420j	Baked chocolate (e.g., Galbo)	2
	D11420k	Small chocolate pieces (e.g., Apollo)	4
	D11423a	Doughnut with a hole	6
	D11423b	Doughnut without a hole	6
	D11425a	Ice cream bar	7
	D11425b	Ice cream cup	6
	D11425c	Iced monaka, iced sandwich	8
	D11425d	Ice cream cone	3
	D11425e	Other ice cream	6
	D11426	Jelly	8
	D11427a	Financier	3
	D11427b	Baumkuchen	5
	D11427c	Pie cookie	3
	D11427d	Brownie and gateau chocolat	6
	D11427e	Swiss roll	5
	D11427f	Apple pie	4
	D11427g	Strawberry sponge cake	5
	D11427h	Cheesecake	6
	D11427i	Cheese tart	4
	D11427j	Strawberry tart	3
	D11427k	Muffin	2
	D11501a	Coffee in a take-away cup	4
	D11501b	Canned coffee	3
	D11502	Black tea ^3^	6
	D11503	Other tea (e.g., barley tea, oolong tea) ^3^	6
	D11504	Water, sugar-free carbonated water, 0 kcal beverage ^3^	6
	D11505	Sports drink ^3^	6
	D11506	Cocoa, Milo ^3^	6
	D11507	Energy drink	4
	D11508	Vegetable juice, green juice ^2^	5
	D11509	Fruit juice ^2^	5
	D11515a	Japanese sake (bottles)	6
	D11515b	Japanese sake (paper cartons)	4
	D11515c	Japanese sake (cup bottles) ^4^	6
	D11516a	Canned beer and happoushu (sparkling alcoholic beverages)	4
	D11516b	Bottled beer and happoushu (sparkling alcoholic beverages)	5
	D11517	Bottled wine	6
	D11519	Japanese shochu (cup bottles) ^4^	6
	D11520a	Bottled sour and chuhai	4
	D11520b	Canned sour and chuhai	5
	D11520c	Plum liquor, in bottles and paper cartons	5
	D11804	Pickled plum	3

^1^ For food items, the four- or five-digit number corresponds to the food code in the Standard Tables of Food Composition in Japan [38]. For dish items, the five-digit number corresponds to minor codes in the dish composition database [39]. Alphabet at the end of each code indicates subdivisions. ^2^ Photographs of commonly used paper cartons were used. ^3^ Photographs of commonly used plastic bottles were used. ^4^ Photographs of commonly used cup bottles were used.

**Table 3:** List of household measurement items photographed.

Items	Number of Selections	Number of Portions for Each Cup/Glass/Spoon
Cylindrical mugs	3	7
Corn-shaped mugs	2	7
Teacups	2	7
Tall glasses	4	7
Short glasses	2	7
Wine glasses	4	7
Sake glasses	3	5 for a small glass and 7 for others
Yunomis (Japanese-style teacups)	5	5 for two small yunomis and 7 for others
Tokkuri (sake bottle)	2	1 (maximum volume)
Choko (small cup)	2	1 (maximum volume)
Measuring spoons	2	1 (maximum volume)
Meal spoons	3	1 (maximum volume)

## Data Availability

The data presented in this study are available on reasonable request from the corresponding author.
